# Antioxidant Defense during Recovery of Resurrection Plant *Haberlea rhodopensis* from Drought- and Freezing-Induced Desiccation

**DOI:** 10.3390/plants11020175

**Published:** 2022-01-10

**Authors:** Gergana Mihailova, Ivanina Vasileva, Liliana Gigova, Emiliya Gesheva, Lyudmila Simova-Stoilova, Katya Georgieva

**Affiliations:** 1 Laboratory of Photosynthesis–Activity and Regulation, Institute of Plant Physiology and Genetics, Bulgarian Academy of Sciences, Acad. G. Bonchev Str., Bl. 21, 1113 Sofia, Bulgaria; gmihailova@bio21.bas.bg; 2 Laboratory of Experimental Algology, Institute of Plant Physiology and Genetics, Bulgarian Academy of Sciences, Acad. G. Bonchev Str., Bl. 23, 1113 Sofia, Bulgaria; ivanina_vasileva1@abv.bg (I.V.); gigova01@gmail.com (L.G.); 3 Laboratory of Plant-Soil Interactions, Institute of Plant Physiology and Genetics, Bulgarian Academy of Sciences, Acad. G. Bonchev Str., Bl. 25, 1113 Sofia, Bulgaria; emiliagesheva@abv.bg; 4 Laboratory of Regulation of Gene Expression, Institute of Plant Physiology and Genetics, Bulgarian Academy of Sciences, Acad. G. Bonchev Str., Bl. 21, 1113 Sofia, Bulgaria; lsimova@mail.bg

**Keywords:** drought stress, low temperature, rehydration, ascorbate, glutathione, antioxidant enzymes

## Abstract

In this study, the contribution of nonenzymatic (ascorbate, glutathione) and enzymatic antioxidants (superoxide dismutase, catalase, glutathione reductase, glutathione S-transferase) in the first hours of recovery of the resurrection plant *Haberlea rhodopensis* from drought- and freezing-induced desiccation was assessed. The initial stage of recovery after desiccation is critical for plants, but less investigated. To better understand the alterations in the activity of antioxidant enzymes, their isoenzyme patterns were determined. Our results showed that ascorbate content remained high during the first 9 h of rehydration of desiccated plants and declined when the leaves′ water content significantly increased. The glutathione content remained high at the first hour of rehydration and then strongly decreased. The changes in ascorbate and glutathione content during recovery from drought- and freezing-induced desiccation showed great similarity. At the beginning of rehydration (1–5 h), the activities of antioxidant enzymes were significantly increased or remained as in dry plants. During 7–24 h of rehydration, certain differences in the enzymatic responses between the two plant groups were registered. The maintenance of a high antioxidant activity and upregulation of individual enzyme isoforms indicated their essential role in protecting plants from oxidative damage during the onset of recovery.

## 1. Introduction

Desiccation-tolerant or resurrection plants, which can survive desiccation to an air-dry state, are an excellent model for studying the mechanisms of drought resistance. Desiccation tolerance is a complex trait achieved through a combination of morphological, physiological, biochemical, molecular, and metabolic changes to prevent lethal cellular damage [1,2,3]. Understanding the mechanisms of how resurrection plants cope with extreme water loss in their vegetative tissues is of great importance for enhancing stress tolerance in crop plants [4]. In order to survive desiccation and fully recover, desiccation-tolerant organisms should overcome two main concomitant stresses, mechanical and oxidative stress. Resurrection plants can avoid the detrimental effect of mechanical stress caused by cell shrinkage during dehydration through increased vacuolation and cell wall folding [5,6]. The replacement of the large central vacuole with many smaller vacuoles reduces the extent of plasmalemma withdrawal from the cell wall during desiccation [7], while the extensive folding of the cell wall is important to maintain cell integrity [8]. Desiccation is associated with metabolic disturbances, leading to the accumulation of reactive oxygen species (ROS). Resurrection plants have evolved two different mechanisms to maintain homeostasis between the generation and quenching of ROS: homoiochlorophylly and poikilochlorophylly [3]. Homoiochlorophyllous desiccation-tolerant plants keep most of their chlorophyll content and maintain the photosynthetic apparatus during desiccation, while in poikilochlorophyllous plants chlorophyll is degraded and thylakoids are dismantled during desiccation and regenerated during rehydration. Photosynthesis is very sensitive to water deficit. Downregulation of photosynthesis during desiccation results in an imbalance between primary and secondary photosynthetic reactions, leading to increased ROS production [9,10]. Overproduced ROS react with cellular macromolecules, causing oxidative damage and disruption of cell functions [1], but ROS are also important signaling molecules that trigger the upregulation of protective mechanisms [11].

Under physiological conditions, ROS are efficiently scavenged by the antioxidant defense system, preventing cellular damages. Accumulation of nonenzymatic antioxidants, such as carotenoids, ascorbate, tocopherols, and glutathione, and enhanced activity of antioxidant enzymes, such as superoxide dismutase (SOD), catalases (CATs), peroxidases (PODs), and glutathione reductase (GR), have been observed during stress conditions [8,12]. Ascorbate and glutathione, as well as related enzymes, have been shown to play a significant role in early plant stress responses to drought [13], but stress-induced changes in the different antioxidants are species specific and depend on the severity and duration of stress [14,15]. Under water-deficient conditions, both drought-resistant and drought-sensitive plants showed the ability to activate their antioxidant system, where the degree of activation and which enzymes are upregulated are crucial [11]. Moreover, high antioxidant capacity in the sensitive plants was observed under moderate drought stress [16]. An increased level of antioxidants in dehydrated leaves of desiccation-tolerant *Sporobolus stapfianus* but not in those of desiccation-sensitive *Sporobolus pyramidalis* was also reported [17]. Under severe drought conditions, the scavenging system in desiccation-sensitive plants becomes saturated, and damage is inevitable [18]. Thus, drought tolerance depends on the capacity of the antioxidant system in vascular plants [9,19]. In contrast to desiccation-sensitive plants, desiccation-tolerant plants can maintain the antioxidant activity in the desiccated state. Farrant et al. [20] showed that the antioxidant enzymes eliminate ROS even at RWC lower than 10%, suggesting that they are well protected under these conditions and their native structure is preserved. Resurrection plants are exposed to the repeated desiccation–rehydration cycle; thus keeping antioxidant activity is necessary for their survival. Kranner et al. [9] followed the antioxidant status of the homoiochlorophyllous resurrection plant *Myrothamnus flabellifolia* after different periods of drought. Upregulation of antioxidants upon rehydration was only observed in vegetative tissues desiccated for 4 months. Further desiccation led to an increase in oxidative damage. After 8 months of desiccation, irreversible leaf damage occurred, which correlated with the breakdown of the antioxidant system. Thus, it was shown that longevity in the desiccated state is limited and that the inability of plants to recover after prolonged desiccation is associated with a lack of functioning antioxidant protection [9,21]. 

It is generally considered that desiccation-tolerant plants must limit the damage during desiccation, maintain physiological integrity in the dry state, and repair the damage upon rehydration [3,22]. Thus, in order to understand the complex strategy of resurrection plants to survive desiccation to an air-dry state, the study of defense mechanisms both during drought and during rehydration is equally important. Over the last few decades, research has focused on protective mechanisms, mainly during desiccation and rarely in the course of rehydration. In fact, some attention has been paid to the later stages of rehydration, when plants regain most of their water content. Rapid water uptake upon rehydration is dangerous for plants as it could induce cellular damages [23]. Hence, the early stage of rehydration of dry plants is potentially harmful, and plants need efficient protection during this period [24,25]. As with desiccation, increased ROS production was observed during rehydration [26,27], and it was shown that more injuries occur during rehydration compared with desiccation due to oxidative stress [28]. That is why the stress of rehydration requires appropriate protective cellular responses [3].

*Haberlea rhodopensis* Friv. is a homoiochlorophyllous resurrection plant with high ecological plasticity. It prefers shady places with high humidity, but can be rarely found in open places exposed to full sunlight [29]. In addition, it possesses high temperature resistance [30]. Unlike most resurrection plants, *H. rhodopensis* is subjected to freezing temperatures in the winter. Temperatures below −6 °C induce desiccation of plants, and they endure the harsh winter conditions in the desiccated state [31]. 

Until now, our studies have focused mainly on the response of *H. rhodopensis* to dehydration triggered by drought or freezing temperatures and the full recovery of plants [29,30,31]. Recently, we investigated for the first time the restoration of photosynthetic activity in the first hours of rehydration of *H. rhodopensis* and found some differences in the response of plants rehydrated after desiccation due to drought and freezing. Plants recovered after freezing-induced desiccation (RAF) regain their RWC more quickly during rehydration compared with plants recovered after drought-induced desiccation (RAD). Additionally, PSI activity is recovered more rapidly in RAF plants, while PSII activity is recovered faster in RAD plants [25]. 

The aim of the present study was to evaluate and compare the role of antioxidant defense during the recovery of the resurrection plant *H. rhodopensis* from drought- and freezing-induced desiccation. We hypothesize that nonenzymatic (reduced and oxidized forms of ascorbate and glutathione) and enzymatic antioxidants (superoxide dismutase, catalase, glutathione reductase, glutathione S-transferase) will make an important contribution to the recovery of dry plants in the first hours of water uptake, when the most significant changes in metabolism are expected. For better understanding of the changes in antioxidant enzymes, their isoenzyme patterns were determined using native polyacrylamide gel electrophoresis (nPAGE) and subsequent staining for enzyme activity in a gel. Considering our previous results [25], some variations in the responses of both plant groups upon rehydration could be expected.

## 2. Results

### 2.1. Ascorbate Content during Rehydration

**Figure 1:**
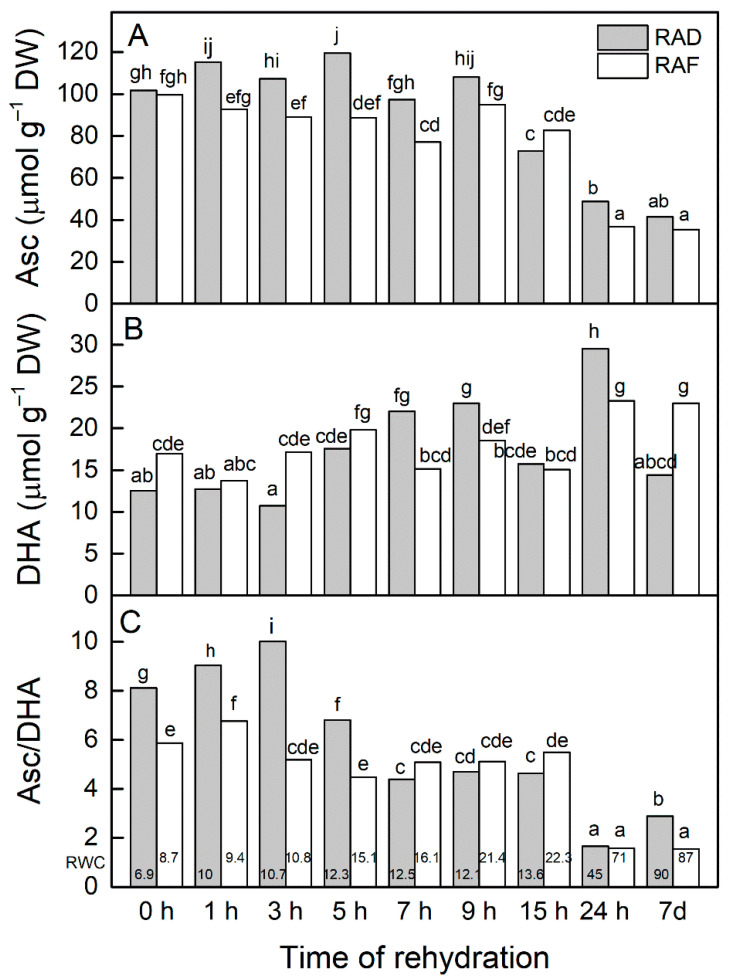
Changes in ascorbate (Asc, **A**) and dehydroascorbate contents (DHA, **B**) and the Asc/DHA ratio (**C**) during the recovery of *Haberlea rhodopensis* from drought- and freezing-induced desiccation. The measurements were performed on air-dried plants (0 h) and after 1, 3, 5, 7, 9, 15, and 24 h as well as 7 days of rehydration after drought- (RAD) and freezing-induced desiccation (RAF). The RWCs of the plants at each time point, presented in %, are shown at the bottom of the columns. Data represent the mean ± SE of *n* = 9. The same letters within a graph indicate no significant differences assessed by the Fisher LSD test (*p* ≤ 0.05) after performing ANOVA.

Recently, we showed that Asc and GSH contents increased during the desiccation of *H. rhodopensis*, reaching the maximum in the dry state, indicating their important role in preventing photooxidation at very low RWC [32]. In the present study, we investigated their significance for the recovery of plants in the course of rehydration from drought- and freezing-induced desiccation. The results showed a similar Asc content in plants desiccated due to drought and freezing stress (Figure 1A, 0 h). The amount of Asc remained high for up to 9 h of rehydration of dry plants after drought stress (RAD) and freezing stress (RAF). It slightly declined after 15 h of rehydration, but the most significant reduction in Asc content was observed after 24 h, when RWC was significantly enhanced. High correlation coefficient of Pearson (*r* = 0.911) was determined for the changes in Asc content in RAD and RAF plants. However, taken together, the values of Asc during rehydration of RAF plants were lower than those of RAD plants. The content of DHA started to increase after 5 h of rehydration of the RAD plants, reaching the maximum after 24 h (Figure 1B). During the rehydration of the RAF plants, the DHA level remained high, and it was higher than that of the RAD plants during the first 5 h of rehydration. The Asc/DHA ratio increased during the first 3 h of rehydration of the RAD plants and after 1 h of rehydration of the RAF plants (*p* ≤ 0.05) compared with the dry plants (Figure 1C). Then, its values declined but were kept at a similar level up to 15 h of rehydration, followed by a strong reduction after 24 h of rehydration. A relatively high Pearson correlation coefficient (*r* = 0.760) was found for the change in the Asc/DHA ratio during the rehydration of both plant groups.

### 2.2. Glutathione Content during Rehydration

**Figure 2:**
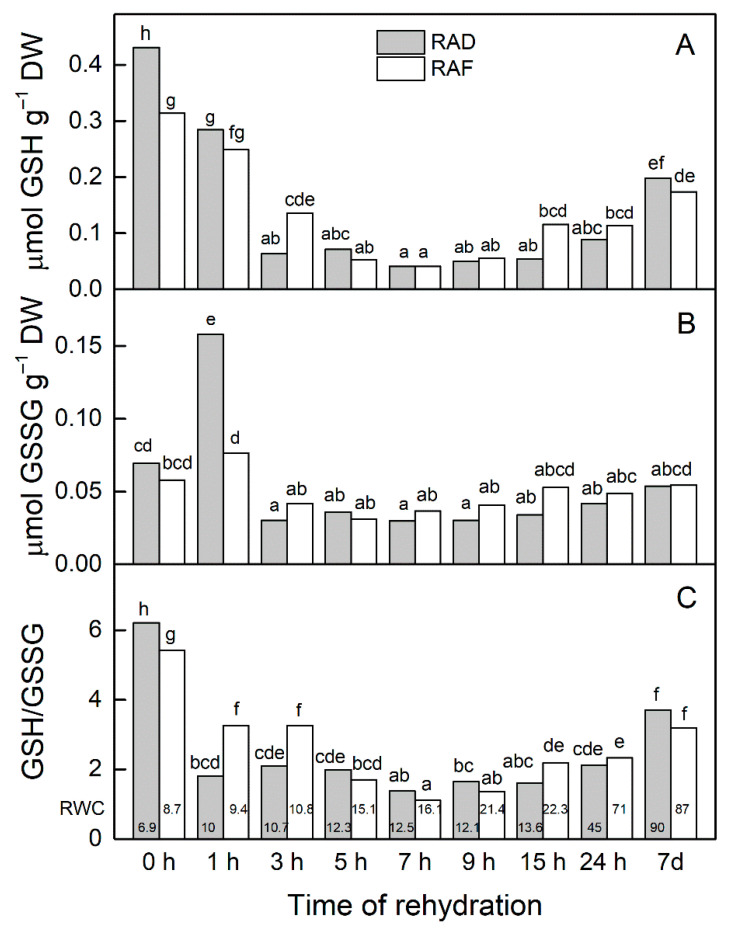
Changes in the contents of reduced (GSH, **A**) and oxidized (GSSG, **B**) glutathione as well as in the GSH/GSSG ratio (**C**) during the recovery of *Haberlea rhodopensis* from drought- and freezing-induced desiccation. The measurements were performed on air-dried plants (0 h) and after 1, 3, 5, 7, 9, 15, and 24 h as well as 7 days of rehydration after drought- (RAD) and freezing-induced desiccation (RAF). The RWCs of the plants at each time point, presented in %, are shown at the bottom of the columns. Data represent the mean ± SE of *n* = 9. The same letters within a graph indicate no significant differences assessed by the Fisher LSD test (*p* ≤ 0.05) after performing ANOVA.

The content of GSH was similarly affected during the rehydration of the RAD and RAF plants (*r* = 0.946). The results presented in Figure 2A show that the content of GSH was kept relatively high 1 h after the rehydration of the plants and then strongly decreased. Similar changes were observed in the content of GSSG (Figure 2B). The enhancement of GSSG after 1 h of rehydration, especially in the RAD plants, was followed by a significant reduction in its amount in the course of the rehydration (*p* ≤ 0.05; Figure 2B). Rehydration led to a strong decline in the GSH/GSSG ratio even at the first hours of rehydration, and it was stronger in the RAD plants (Figure 2C). This ratio reached minimum values after 7 h of rehydration, after which there was a tendency for its increase, and after 7 d of rehydration, the values were significantly higher compared with those after 24 h in both plant groups (*p* ≤ 0.05). In fact, a similar tendency was detected for the changes in GSH and GSSG.

### 2.3. SOD Activity during Rehydration

**Figure 3:**
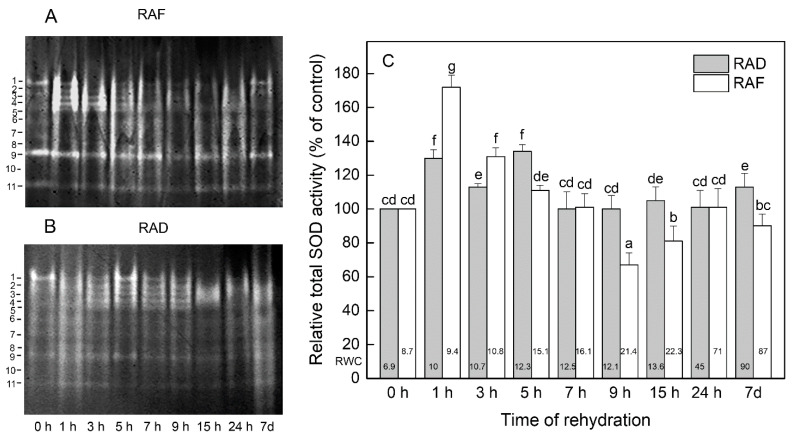
Isoenzyme patterns (**A**,**B**) and relative total activity (**C**) of superoxide dismutase (SOD) during the recovery of *Haberlea rhodopensis* from drought- and freezing-induced desiccation. The SOD isoenzymes are numbered from cathode to anode. The total activity for a particular treatment is expressed as percentage of control. The analyses were performed with air-dried plants (0 h) and after 1, 3, 5, 7, 9, 15, and 24 h as well as 7 days of rehydration after drought- (RAD) and freezing-induced desiccation (RAF). The RWCs of the plants at each time point, presented in %, are shown at the bottom of the columns. Data represent the mean ± SE of *n* = 3. The same letters within a graph indicate no significant differences assessed by the Fisher LSD test (*p* ≤ 0.05) after performing ANOVA.

Besides the changes in the amount of Asc and GSH, the activities of some antioxidant enzymes during the rehydration of the RAD and RAF plants were investigated. In all *H. rhodopensis* leaf protein extracts, 11 SOD isoenzymes (numbered in the order of increasing electrophoretic mobility) were resolved in gels (Figure 3A,B). During the initial 1–5 h of recovery of the plants from drought- and freezing-induced desiccation, a significant increase (*p* ≤ 0.05) in the relative total activity of the enzyme was observed (Figure 3C), mainly due to the activation of slower-moving isoforms 2–5 (Figure 3A,B). With increasing rehydration time, the SOD activity in the RAF plants decreased and remained relatively constant after 24 h and 7 d, when the RWCs of the plants were almost completely restored (Figure 3C). Although the prolonged rehydration decreased (9 and 15 h) or did not have a strong effect (7 h, 24 h, and 7 d) on the total activity, specific SOD isoenzymes were upregulated (Figure 3A). For example, the activities of isoenzymes 8 and 11 were increased by about 33% and 43%, respectively, after 15 h of rehydration compared with the desiccated plants (0 h), while isoenzyme 3, 4, 6, and 8 activities were increased by about 400%, 62%, 136%, and 23% (*p* ≤ 0.05), respectively, after 24 h. The rehydration of the RAD plants for 7 to 24 h did not affect the total SOD activity, but isoenzymes 2, 3, 4 (except for 24 h), and 8 (except for 7 h) were upregulated (*p* ≤ 0.05; Figure 3B). Upon complete rehydration of these plants (7 d), the total SOD activity was 13% higher (*p* ≤ 0.05) than that of the dry plants (Figure 3C) due to the activation of isoenzymes 3 (228%), 4 (149%), 6 (45%), 8 (603%), 9 (46%), and 11 (174%) (Figure 3B). 

### 2.4. CAT Activity during Rehydration

**Figure 4:**
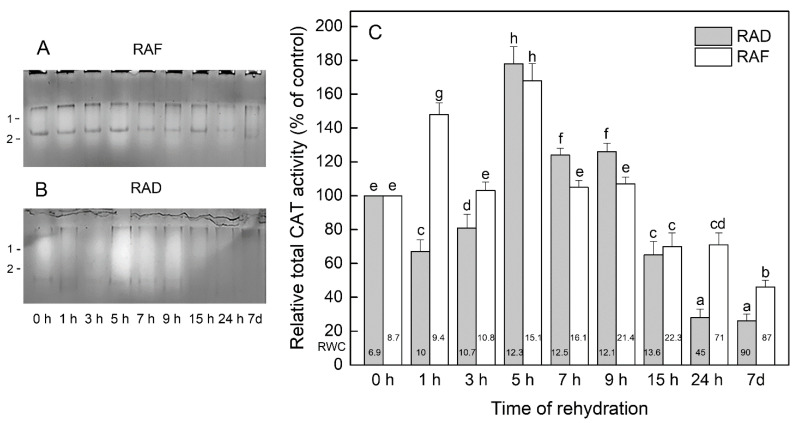
Isoenzyme patterns (**A**,**B**) and relative total activity (**C**) of catalase (CAT) during the recovery of *Haberlea rhodopensis* from drought- and freezing-induced desiccation. The CAT isoenzymes are numbered from cathode to anode. The total activity for a particular treatment is expressed as percentage of control. The analyses were performed with air-dried plants (0 h) and after 1, 3, 5, 7, 9, 15, and 24 h as well as 7 days of rehydration after drought- (RAD) and freezing-induced desiccation (RAF). The RWCs of the plants at each time point, presented in %, are shown at the bottom of the columns. Data represent the mean ± SE *n* = 3. The same letters within a graph indicate no significant differences assessed by the Fisher LSD test (*p* ≤ 0.05) after performing ANOVA.

Staining for CAT showed two activity bands in all *H. rhodopensis* samples (Figure 4A,B). In general, isoenzyme 1 was more active than isoenzyme 2, but both isoenzymes were responsive to the treatments. In the RAF plants (Figure 4A), the activity of isoenzyme 1 was 41% and 35% higher after 1 and 5 h of rehydration, respectively, and that of isoenzyme 2 was 68% and 163% higher than that of the dry plants (0 h; *p* ≤ 0.05), leading to a significant increase in the total CAT activity by 48% and 68%, respectively (Figure 4C). During the following time points of rehydration, the enzyme activity gradually decreased, reaching the lowest values in the fully recovered plants after 7 d (Figure 4C). The relative total CAT activity of the plants rehydrated for 3, 7, and 9 h was similar to that of the dry plants, but the activity of their isoenzyme 2 was about 34%, 43%, and 85% higher (*p* ≤ 0.05) (Figure 4A,C). The RAD plants showed significantly increased total CAT activity (178%, 124%, and 126%, respectively) after 5, 7, and 9 h of rehydration compared with the dry plants and decreased enzyme activity (*p* ≤ 0.05) during all other rehydration time points (Figure 4C), both isoenzymes being responsible for these changes (Figure 4B).

### 2.5. GR Activity during Rehydration

**Figure 5:**
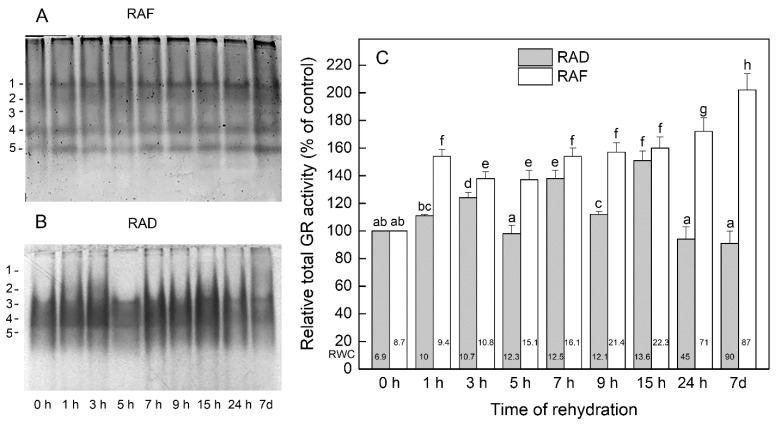
Isoenzyme patterns (**A**,**B**) and relative total activity (**C**) of glutathione reductase (GR) during the recovery of *Haberlea rhodopensis* from drought- and freezing-induced desiccation. The GR isoenzymes are numbered from cathode to anode. The total activity for a particular treatment is expressed as percentage of control. The analyses were performed with air-dried plants (0 h) and after 1, 3, 5, 7, 9, 15, and 24 h as well as 7 days of rehydration after drought- (RAD) and freezing-induced desiccation (RAF). The RWCs of the plants at each time point, presented in %, are shown at the bottom of the columns. Data represent the mean of ± SE *n* = 3. The same letters within a graph indicate no significant differences assessed by the Fisher LSD test (*p* ≤ 0.05) after performing ANOVA.

The isoenzyme profile of GR of *H. rhodopensis* was represented by five isoforms (Figure 5A,B). The total GR activity was significantly stimulated in the course of the rehydration of the RAF plants (Figure 5C). Enzyme activity was 54% higher than in the dry plants after 1 h of rehydration (*p* ≤ 0.05), reaching the maximum (200%) after 7 d. All GR isoforms contributed to this enhanced total activity, especially isoforms 1, 3, and 5 (Figure 5A). In the RAD plants, the total activity of GR increased by 11%, 24%, 38%, 12%, and 51% (*p* ≤ 0.05) compared with the dry plants after 1, 3, 7, 9, and 15 h of rehydration, respectively, and decreased back to the level in the dry plants after 24 h and 7 d of rehydration (Figure 5C). However, the activity of isoform 1 was 152% and 323% higher after 24 h and 7 d of rehydration, respectively, and isoform 5 was about 34% more active than in the dry plants after 24 h of rehydration (Figure 5B).

### 2.6. GST Activity during Rehydration

**Figure 6:**
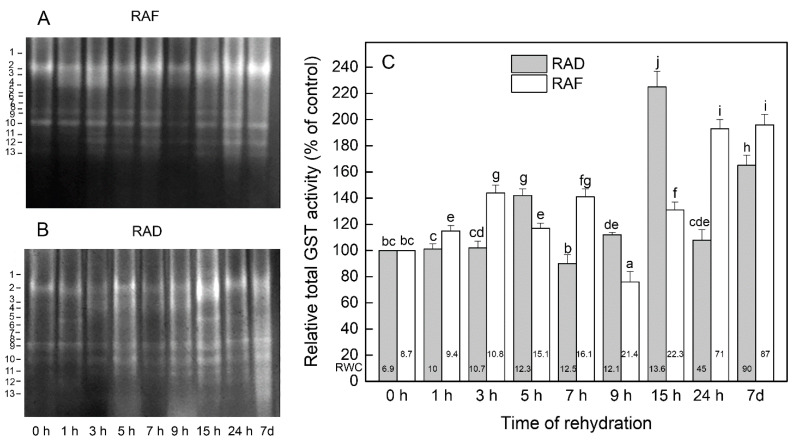
Isoenzyme patterns (**A**,**B**) and relative total activity (**C**) of glutathione S-transferase (GST) during the recovery of *Haberlea rhodopensis* from drought- and freezing-induced desiccation. The GST isoenzymes are numbered from cathode to anode. The total activity for a particular treatment is expressed as percentage of control. The analyses were performed with air-dried plants (0 h) and after 1, 3, 5, 7, 9, 15, and 24 h as well as 7 days of rehydration after drought- (RAD) and freezing-induced desiccation (RAF). The RWCs of the plants at each time point, presented in %, are shown at the bottom of the columns. Data represent the mean ± SE of *n* = 3. The same letters within a graph indicate no significant differences assessed by the Fisher LSD test (*p* ≤ 0.05) after performing ANOVA.

Thirteen GST isoenzymes were distinguished in gels (Figure 6A,B). Similar to GR, the total GST activity during the rehydration of the RAF plants was higher than that of the desiccated plants (*p* ≤ 0.05; Figure 6C). The most pronounced increase in its activity was observed in completely rehydrated plants (by 96%) and after 24 h of rehydration (by 93%), which was related to the upregulation of all isoenzymes. The activities of all GST isoenzymes were also significantly stimulated after 3 h (except isoenzyme 2) and after 7 h of rehydration, but to a lesser extent (Figure 6A). Although the total GST activity declined by about 24% (*p* ≤ 0.05) after 9 h of rehydration, the activities of isoenzymes 4 and 12 were increased by about two times (Figure 6A,C). Rehydration of the RAD plants for 5, 9, and 15 h and 7 d resulted in a significant increase in relative total GST activity of about 42%, 12%, 125%, and 65%, respectively, compared with the desiccated plants (Figure 6C). Different isoenzymes participated in this increase to varying degrees (Figure 6B). After 5 h of rehydration, for example, the activities of isoforms 3, 4, 5, 10, and 11 increased by 118%, 111%, 70%, 170%, and 169%, respectively, compared with the dry plants (*p* ≤ 0.05), while after 7 d the fastest-moving isoforms (10–13) had a major contribution. The highest total GST activity in plants rehydrated for 15 h was due to the activation of all isoforms, but mostly due to the enhanced activities of isoforms 1, 2, 5, 10, and 11 (2.3, 2.1, 3.3, 3.5, and 3.8 times, respectively). The total GST activity did not change significantly after 1, 3, 7, and 24 h of rehydration compared with the dry plants, but some specific isoenzymes were activated in response to the treatments. The most responsive after 1 h of rehydration was isoenzyme 5 (83% higher activity), while isoenzymes 3 and 4 had increased activities after 3 h (by 65% and 79%, respectively) and 7 h (by about 30% each), and isoenzymes 10 and 11 were by about 60% and 167%, respectively, more active after 24 h of rehydration.

## 3. Discussion

Maintaining high antioxidant activity in the desiccated state is a distinguishing feature of resurrection plants. These antioxidants can help them to survive in the air-dry state and afterwards during rehydration. In fact, antioxidants accumulated upon desiccation are thought to constitute a reserve that can be used during the early stages of rehydration to protect against ROS, overproduced in the process of metabolism reconstitution, thus helping the plants to recover [8,33]. We previously showed that drought stress increased the contents of Asc and GSH as well as the activities of APX, GR, and GST, and they reached the maximum in air-dried *H. rhodopensis* leaves [32]. In addition, our recent studies demonstrated that the activities of the antioxidant enzymes SOD, CAT, GR, and GST remained high during freezing stress and freezing-induced desiccation, indicating their important role in overcoming oxidative stress under these conditions [34]. However, as in most other studies on resurrection plants, we have so far investigated the changes in antioxidants status in completely recovered plants. Taking into account that the first hours of rehydration are critical and decisive for the recovery of plants, we now explored the role of antioxidant defense during this period. It should be noted that rehydration is a time-consuming process, and although RWC was significantly increased and photochemical activity was restored after 24 h, complete recovery of *H. rhodopensis* was achieved after 7 d [25]. Our present results showed that Asc content remained high during the first 9 h of rehydration of dry *H. rhodopensis* plants, and a significant reduction of its amount was observed with increasing RWC and photosynthetic activity of the plants (Figure 1). Similarly, the Asc/DHA ratio strongly declined after 24 h. It is proposed that high ascorbate content was maintained during desiccation and early rehydration by a combination of de novo synthesis and regeneration of ascorbate by ascorbate peroxidase [8]. Ascorbate has multiple physiological roles. Together with GSH, it participates in the ascorbate–glutathione cycle, which plays an important role in overcoming oxidative stress during the desiccation of *H. rhodopensis* we recently demonstrated [32]. As a powerful antioxidant, Asc can directly scavenge ROS and act as an electron donor for ROS-detoxifying enzymes [35]. It has been shown that Asc is a cofactor for violaxanthin de-epoxidase [36]. In addition, it has been suggested that a low ascorbate level could significantly limit the excitation energy dissipation in vivo [37]. Indeed, the dissipation of excess excitation energy under stress conditions is a significant defense mechanism and has been shown to be important in avoiding photooxidation during the first hours of *H. rhodopensis* rehydration [25]. From the present results, it is clear that Asc plays a significant role in the first 1–9 h of *H. rhodopensis* recovery, when the RWC varies between 9.4% and 21.4%. In another resurrection plant, *Ramonda serbica*, it was found that the Asc content decreased significantly after 5 h [38] and 10 h [39] of rewatering compared with the dried leaves. During these early hours of rehydration, however, the RWC levels in *R. serbica* leaves were relatively high, about 32% and 50%, respectively.

The GSH and GSSG contents remained high at the first hour of rehydration of *H. rhodopensis* and then significantly decreased (Figure 2). Similarly, a sharp reduction in GSH content was observed after 5 h [38] and 10 h [39] of rehydration of *R. serbica*. In fact, the amount of GSH measured at 10 h of rehydration of *R. serbica* was about three times lower than at the beginning of rehydration and remained at the same level up to 36 h. The data of Djilianov et al. [40] on in vitro propagated *H. rhodopensis* also showed that the desiccation-induced increase in the contents of GSH and GSSG significantly and sharply decreased during recovery. It was found that GSH is involved in the oxidative stress responses in almost all the resurrection plants studied [9,39,41]. However, in most of the studies the highest level of GSH was determined in desiccated leaves. For example, the GSH content measured after 24 h of rehydration of *Boea hygrometrica* was half of that of desiccated plants, and then it slightly decreased until full turgor was restored [42]. Thus, it was concluded that GSH may play a major role in protecting *B. hygrometrica* from the oxidative stress induced by dehydration. Under our experimental conditions, the values of GSH and the GSH/GSSG ratio were about 50% and 60%, respectively, of those of completely dry plants after 7 d of rehydration when the plants regained their RWC (Figure 2). Many of the functions of GSH are linked to defense [35]. It is a very important soluble antioxidant, a substrate for glutathione peroxidases and glutathione S-transferases. As a precursor to phytochelatins, GSH plays a role in heavy metal detoxification [43], and it may protect the thiol status of proteins against oxidative stress by the formation of a stable disulfide with a protein thiol [44]. It has been suggested that GSH not only acts as an antioxidant during the dehydration–rehydration cycles, but also can synchronize different recovery processes [41]. 

Upregulation of antioxidant enzyme activities has been established to be important for plants’ protection during desiccation induced by drought and freezing stresses [45,46]. Survival in acute dehydration conditions depends on the genotype-specific characteristics of the plants, the intensity and duration of stress, and the speed and efficiency of plant recovery after rehydration [11]. While most studies have focused on elucidating the role of the plant’s enzyme antioxidant system during desiccation, the response of antioxidant enzymes in the time course of rehydration has been less studied.

At the beginning of rehydration (1–5 h) of both RAF and RAD *H. rhodopensis* plants, the activities of SOD, CAT, GR, and GST were significantly increased or remained as high as in dry plants. It was shown that during the first few hours of rehydration, when limited water prevents full physiological activity but allows adverse reactions associated mainly with excessive ROS generation to proceed, a wide range of drought-resistant plants activate their antioxidant enzymes to overcome this critical and least stable period [24,28,47,48]. Jovanović et al. [48] found a significant increase in CAT, GR, and ascorbate peroxidase (APX) activities during the first 6–12 h of rehydration of *Ramonda nathaliae* and weak SOD activation during the first 6 h. Upon rehydration for 24 h, the CAT activity remained high, while those of APX and GR sharply declined but were higher compared with dried plants [48]. However, in *R. serbica*, the changes in antioxidant enzymes were quite different [49]. Rehydration of desiccated *R. serbica* resulted in a remarkable decrease in the activities of SOD, APX, and POD at the first 6 h, and after 24 h of rehydration, enzymes’ activities recovered to those of the dry leaves. 

During 7–24 h of *H. rhodopensis* rehydration, certain differences in the antioxidant enzyme responses of RAF and RAD plants were registered. For example, after 9 h of rehydration, SOD and GST activities decreased significantly and CAT activity was not affected in the RAF plants, whereas in the RAD plants GST and CAT were significantly stimulated and SOD was as active as in the desiccated plants. In fully rehydrated *H. rhodopensis* plants (7 d), the activities of the studied antioxidant enzymes were upregulated or were similar to those in dry plants, except for CAT, which was downregulated. Unlike *H. rhodopensis*, at the end of the rehydration (48 h) of *R. nathaliae* the activities of SOD, CAT, and GR were increased, but the APX activity was as in the desiccated plants [48]; completely rehydrated *R. serbica* (70 h) downregulated its SOD, APX, and POD enzymes [50]; and in *Selaginella brachystachya*, rehydration resulted in significantly lower SOD, CAT, GR, and POD activities compared with those in the desiccated plants [4]. The study of different species of resurrection plants shows that the response of their antioxidant enzymes to rehydration is not universal, and each species has its own specific mechanism of enzymatic antioxidant protection [4,48,49,51,52]. 

## 4. Materials and Methods

### 4.1. Desiccation and Rehydration of Plants

Desiccation and rehydration of plants were performed as described by Georgieva et al. [25]. *H. rhodopensis* tufts collected from the Rhodope Mountains and cultivated in pots with peat soil (Stender, Schermbeck, Germany) under ex situ (outdoor) environmental conditions were used in the studies. Some tufts were transferred to a climatic chamber, FytoScope FS 130 (Photon Systems Instruments, Drásov, Czech Republic), and kept at 25/18 °C day/night temperature, 60% humidity, 12 h photoperiod, and an irradiance of 25 μmol (photon) m^–2^ s^–1^ for 2 weeks. The plants were then subjected to drought stress by stopping irrigation until they reached an air-dry state. The other tufts were left outdoor (light intensity of 30–60 μmol (photon) m^–2^ s^–1^), where they were exposed to cold and freezing temperatures in natural conditions during autumn and winter (November 2018–February 2019). When the temperature dropped to about −10 °C, the dehydration of the plants began, and they overwintered in an air-dry state. The rehydration of the plants after drought- and freezing-induced desiccation was carried out in laboratory conditions at 21–23 °C and a light intensity of 25–30 μmol (photon) m^–2^ s^–1^. Initially, the soil was well watered, and then the pots were placed in a modified desiccator, where constant high humidity was provided by a water pump. Measurements were conducted on dry leaves (0 h) and after 1, 3, 5, 7, 9, 15, and 24 h and 7 d of rehydration.

### 4.2. Determination of Relative Water Content (RWC)

The RWC of *H. rhodopensis* leaves was assessed gravimetrically by measuring their weight before and after drying in an oven at 80 °C to a constant mass. Before oven drying, the saturated weight was measured on leaf disks maintained for 12–16 h at 4 °C in the dark floating on water. The results were expressed as the percentage of water content in dehydrated tissue compared with water-saturated tissues using the equation:

RWC (%) = (fresh weight − dry weight) × 100/(saturated weight − dry weight)

### 4.3. Determination of Ascorbate (Asc) and Dehydroascorbate (DHA)

Asc content was determined according to Kampfenkel et al. [53]. An amount of 100 mg of leaf tissue was ground with 2 mL cold 6% trichloroacetic acid (TCA) and centrifuged at 15,000× *g* for 20 min at 4 °C. To 0.2 mL of the supernatant, 0.6 mL 0.2 M K-phosphate buffer (pH 7.4), 0.2 mL dd H_2_O, 1 mL 10% TCA, 0.8 mL 42% H_3_PO_4_, 0.8 mL 4% 2,2′-dipyridyl, and 0.4 mL 3% FeCl_3_ were added. To determine the content of DHA, the total ascorbate (tAsc) content was measured as follows: 0.2 mL of the supernatant was mixed with 0.2 mL of 10 mM dithiothreitol (DTT), and the samples were incubated for 15 min at 42 °C, followed by the addition of 0.2 mL of 0.5% N-ethylmaleimide (NEM). During this incubation, DHA is reduced to Asc by DTT, and excess DTT is then removed by NEM. The reaction mixtures were prepared as described above for Asc. All samples were incubated for 40 min at 42 °C, and their absorbance was read at 525 nm using a UV–VIS T70 spectrophotometer (PG Instruments, UK). The DHA content was calculated as the difference between tAsc and Asc.

### 4.4. Determination of Total Glutathione and Glutathione Disulfide

The method of Griffith [54] was used to determine the contents of total glutathione (GSH + GSSG) and glutathione disulfide (GSSG). An amount of 100 mg of leaf material was extracted with 2 mL cold 5% (w/v) sulfosalicylic acid in 0.1 M K-phosphate buffer (pH 7.6), containing 5 mM EDTANa_2_. The homogenate was centrifuged at 14,000× *g* for 20 min at 4 °C. The reaction mixture for the determination of total glutathione consisted of the obtained supernatant, 0.5 M potassium phosphate buffer (pH 7.6), 0.1 M K-phosphate buffer (pH 7.6) containing 5 mM EDTANa_2_, 3 mM NADPH, 6 mM DTNB, and 40 EU mL^−1^ glutathione reductase. To assay for GSSG, the extract was mixed with 0.5 M K-phosphate buffer (pH 7.6) and 2-vinylpyridine (2-VP). After 1 h, diethyl ether was added, which removed the GSH–2-VP complex and the excess of 2-VP. The aqueous layer was used for the color reaction described above. The increase in the absorbance of all samples was monitored at 412 nm in the linear part of the curve. The GSH content was the difference between total GSH and GSSG.

### 4.5. Protein Extraction, nPAGE, and Antioxidant Enzyme Activity Staining

Protein extraction was performed according to Mladenov et al. [55] with slight modifications. Briefly, after grinding in liquid nitrogen, leaf material (0.5 g FW) was homogenized in ice-cold 50 mM potassium phosphate buffer (PPB, pH 7.8), containing 10 mM KCl, 1 mM EDTA, 1.25 mM PEG 4000, 0.5 M sucrose, 20 mM ascorbic acid, 10 mM dithiothreitol (DTT), 0.1% Triton X-100, 2 mM PMSF, and 2% (*w/v*) Polyclar AT. After centrifugation for 30 min at 15,000× *g*, 4 °C, total protein extracts were desalted on Sephadex G-25 mini columns. Eluted proteins were supplemented with sucrose (20% final concentration, *w/v*) and stored in aliquots at −80 °C. The protein concentration was calculated following the dye-binding assay [56]. Equal amounts of protein (15 µg) from the leaves of plants exposed to different treatments were subjected to discontinuous PAGE under nondenaturing, nonreducing conditions, as described by Laemmli [57], but omitting SDS. Electrophoretic separation of proteins lasted 4–5 h at a constant current of 35 mA per gel. When nPAGE was finished, separate gels were stained for the activities of superoxide dismutase (SOD, EC 1.15.1.1), catalase (CAT, EC 1.11.1.6), glutathione reductase (GR, EC 1.6.4.2), and glutathione S-transferase (GST, EC 2.5.1.18). To visualize the bands with SOD activity, gels (10% polyacrylamide) were soaked in 0.1 mM nitroblue tetrazolium (NBT), 0.05 mM riboflavin, and 0.3% (*v/v*) tetramethyl ethylene diamine in 50 mM PPB (pH 7.8) for 20 min in the dark. Thereafter, the gels were submerged in dH_2_O and exposed to a light box for about 10 min [58]. For CAT activity staining, a method described by Chandlee and Scandalios [50] was applied. The gels (6.5%) were pretreated in 0.01% H_2_O_2_ for 10 min and incubated in a staining solution containing 1% (*w/v*) ferric chloride and 1% (*w/v*) potassium ferricyanide mixed in equal volumes during use. The staining solution for GR isoenzyme pattern and activity determination consisted of 0.24 mM 3-(4,5-dimethylthiazol-2-yl)-2,5-diphenyltetrazolium bromide (MTT), 0.34 mM 2,6-dichlorophenolindophenol, 3.6 mM GSSG, and 0.4 mM NADPH in 250 mM Tris-HCl buffer (pH 7.8). According to Anderson et al. [59], 7.5% gels were immersed in this solution for 1 h in darkness. GST isoforms and activities were detected as described by Ricci et al. [60]. Briefly, the 10% resolved polyacrylamide gels, equilibrated in 100 mM PPB (pH 6.5) for 10 min, were transferred to a reaction mixture composed of 4.5 mM GSH, 1 mM 1-chloro-2,4-dinitrobenzene (CDNB), and 1 mM NBT in the same PPB buffer at 37 °C for 10 min. Further, the gels were incubated at room temperature in 100 mM Tris-HCl (pH 9.6) containing 3 mM phenazine methosulfate (PMS) until they became uniformly blue, while the bands with GST activity were achromatic. After staining, the enzyme patterns were documented using the UVItec gel documentation system (Cambridge, UK) and analyzed using Gel-Pro32 Analyzer software (Media Cybernetics, Rockville, MD, USA). The intensity (activity) of each band (isoenzyme) resolved was measured as total integrated optical density (IOD) in arbitrary units. Each enzyme had more than one isoenzyme, and the sum of their IOD values was considered total enzyme activity for a particular treatment. The experiments to determine the pattern and activity of each enzyme were repeated at least three times. For easier comparison, the values for total enzyme activity and for the activity of the individual isoforms of each enzyme of the desiccated plants were tentatively assumed to be 100%, and the corresponding values of the rehydrated plants were calculated relative to those of the desiccated ones.

### 4.6. Statistical Analysis

The rehydration of desiccated plants as a result of both drought and freezing stress was repeated twice. At each time point, leaves from 6 different tufts were collected, and their mean samples were used for biochemical studies. For the determination of the contents of nonenzymatic antioxidants, 3 biological replicates (mean samples from 6 different tufts) and 3 technical replicates (*n* = 9) were used. The experiments to determine the pattern and activity of each enzyme were repeated at least 3 times (*n* = 3). Comparison of means was performed by the Fisher least significant difference (LSD) test at *p* ≤ 0.05 following ANOVA. A statistical software package (Statgraphics Plus, version 5.1 for Windows, USA) was used. Pearson’s correlation coefficient (*r*), calculated in Microsoft Excel, was used to measure the strength of a linear association between two variables. The formulas return a value between –1 and 1, where 1 indicates a strong positive relationship and –1 indicates a strong negative relationship. A result of zero indicates no relationship at all. 

## 5. Conclusions

In the present study, the contribution of selected nonenzymatic and enzymatic antioxidants at different time points of the recovery process of the resurrection plant *H. rhodopensis* from drought- and freezing-induced desiccation was identified. The high activities of SOD, CAT, GR, and GST, together with the increased level of ascorbate and glutathione confirmed our hypothesis about the crucial importance of enzymatic and nonenzymatic antioxidant protection in the first hours of rehydration of desiccated plants. In fact, while the Asc content remained high, up to 9 h of rehydration, the GSH level sharply declined at the third hour of recovery. With prolonged rehydration, enzymatic antioxidant protection ensured successful plant recovery, although in RAD and RAF plants different enzymes played a major role. Moreover, at the end of rehydration CAT, GR, and GST showed higher activities in RAF plants compared with RAD plants. In general, the activities of the studied antioxidant enzymes of *H. rhodopensis* remain high in the process of plant rehydration after desiccation. All isoenzymes, or at most time points of rehydration-specific isoenzymes, were responsible for this high total enzyme activity. Even in the few cases of decreased total enzyme activity, the activities of specific isoenzymes were significantly increased. The maintenance of a high total activity of antioxidant enzymes and/or the upregulation of individual enzyme isoforms upon rehydration of *H. rhodopensis* indicated the important role of both mechanisms in protecting plants from oxidative damage during the recovery of normal metabolic functions.

## Data Availability

Data are contained within the article.

## References

[1] Colville L., Kranner I. (2010). Desiccation tolerant plants as model systems to study redox regulation of protein thiols. Plant Growth Regul..

[2] Shivaraj Y.N., Plancot B., Gügi B., Vicré-Gibouin M., Driouich A., Govind S.R., Devaraja A., Kambalagere Y. (2018). Perspectives on structural, physiological, cellular, and molecular responses to desiccation in resurrection plants. Scientifica.

[3] Oliver M.J., Farrant J.M., Hilhorst H.W., Mundree S., Williams B., Bewley J.D. (2020). Desiccation tolerance: Avoiding cellular damage during drying and rehydration. Annu. Rev. Plant Biol..

[4] Shivaraj Y.N., Plancot B., Ramdani Y., Gügi B., Kambalagere Y., Jogaiah S., Driouich A., Govind S.R. (2021). Physiological and biochemical responses involved in vegetative desiccation tolerance of resurrection plant *Selaginella brachystachya*. 3 Biotech.

[5] Moore J.P., Nguema-Ona E., Chevalier L., Lindsey G.G., Brandt W.F., Lerouge P., Farrant J.M., Driouich A. (2006). Response of the leaf cell wall to desiccation in the resurrection plant *Myrothamnus flabellifolius*. Plant Physiol..

[6] Georgieva K., Rapparini F., Bertazza G., Mihailova G., Sárvári É., Solti Á., Keresztes Á. (2017). Alterations in the sugar metabolism and in the vacuolar system of mesophyll cells contribute to the desiccation tolerance of *Haberlea rhodopensis* ecotypes. Protoplasma.

[7] Farrant J.M. (2000). A comparison of mechanisms of desiccation-tolerance among three angiosperm resurrection plant species. Plant Ecol..

[8] Morse M., Rafudeen M., Farrant J.M. (2011). An overview of the current understanding of desiccation tolerance in the vegetative tissues of higher plants. Adv. Bot. Res..

[9] Kranner I., Beckett R.P., Wornik S., Zorn M., Pfeifhofer H.W. (2002). Revival of a resurrection plant correlates with its antioxidant status. Plant J..

[10] Reddy A., Chaitanya K., Vivekanandan M. (2004). Drought-induced responses of photosynthesis and antioxidant metabolism in higher plants. J. Plant Physiol..

[11] Laxa M., Liebthal M., Telman W., Chibani K., Dietz K.-J. (2019). The role of the plant antioxidant system in drought tolerance. Antioxidants.

[12] Pandey V., Ranjan S., Deeba F., Pandey A.K., Singh R., Shirke P.A., Pathre U.V. (2010). Desiccation-induced physiological and biochemical changes in resurrection plant *Selaginella bryopteris*. J. Plant Physiol..

[13] Du B., Rennenberg H. (2018). Physiological responses of lavender (*Lavandula angustifolia* Mill.) to water deficit and recovery. S. Afr. J. Bot..

[14] Chaves M.M., Maroco J.P., Pereira J.S. (2003). Understanding plant responses to drought—from genes to the whole plant. Funct. Plant Biol..

[15] Talbi S., Romero-Puertas M.C., Hernández A., Terrón L., Ferchichi A., Sandalio L.M. (2015). Drought tolerance in a Saharian plant *Oudneya Africana*. Role of antioxidant defences. Environ. Exp. Bot..

[16] Maevskaya S.N., Nikolaeva M.K. (2013). Response of antioxidant and osmoprotective systems of wheat seedlings to drought and rehydration. Russ. J. Plant Physiol..

[17] Oliver M.J., Guo L., Alexander D.C., Ryals J.A., Wone B.W., Cushman J.C. (2011). A sister group contrast using untargeted global metabolomic analysis delineates the biochemical regulation underlying desiccation tolerance in *Sporobolus stapfianus*. Plant Cell.

[18] Mundree S.G., Baker B., Mowla S., Peters S., Marais S., Vander Willigen C., Govender K., Maredza A., Muyanga S., Farrant J.M. (2002). Physiological and molecular insights into drought tolerance. Afr. J. Biotechnol..

[19] Dinakar C., Bartels D. (2013). Desiccation tolerance in resurrection plants: New insights from transcriptome, proteome and metabolome analysis. Front. Plant Sci..

[20] Farrant J.M., Brandt W., Lindsey G. (2007). An overview of mechanisms of desiccation tolerance in selected angiosperm resurrection plants. Plant Stress.

[21] Kranner I., Birtic S. (2005). A modulating role for antioxidants in desiccation tolerance. Integr. Comp. Biol..

[22] Bewley J.D. (1979). Physiological aspects of desiccation tolerance. Annu. Rev. Plant Physiol..

[23] Oliver M.J., Tuba Z., Mishler B.D. (2000). The evolution of vegetative desiccation tolerance in land plants. Plant Ecol..

[24] Rakić T., Lazarević M., Jovanović Ž.S., Radović S., Siljak-Yakovlev S., Stevanović B., Stevanović V. (2014). Resurrection plants of the genus Ramonda: Prospective survival strategies–unlock further capacity of adaptation, or embark on the path of evolution?. Front. Plant Sci..

[25] Georgieva K., Mihailova G., Velitchkova M., Popova A. (2020). Recovery of photosynthetic activity of resurrection plant *Haberlea rhodopensis* from drought- and freezing-induced desiccation. Photosynthetica.

[26] Mayaba N., Minibayeva F., Beckett R.P. (2002). An oxidative burst of hydrogen peroxide during rehydration following desiccation in the moss *Atrichum androgynum*. New Phytol..

[27] Weissman L., Garty J., Hochman A. (2005). Rehydration of the lichen *Ramalina lacera* results in production of reactive oxygen species and nitric oxide and a decrease in antioxidants. Appl. Environ. Microbiol..

[28] Sgherri C.L.M., Loggini B., Bochicchio A., Navari-Izzo F. (1994). Antioxidant system in *Boea hygroscopica*: Changes in response to desiccation and rehydration. Phytochemistry.

[29] Sárvári É., Mihailova G., Solti Á., Keresztes Á., Velitchkova M., Georgieva K. (2014). Comparison of thylakoid structure and organization in sun and shade *Haberlea rhodopensis* populations under desiccation and rehydration. J. Plant Physiol..

[30] Mihailova G., Petkova S., Büchel C., Georgieva K. (2011). Desiccation of the resurrection plant *Haberlea rhodopensis* at high temperature. Photosynth. Res..

[31] Mihailova G., Solti Á., Sárvári É., Keresztes Á., Rapparini F., Velitchkova M., Simova-Stoilova L., Aleksandrov V., Georgieva K. (2020). Freezing tolerance of photosynthetic apparatus in the homoiochlorophyllous resurrection plant *Haberlea rhodopensis*. Environ. Exp. Bot..

[32] Georgieva K., Dagnon S., Gesheva E., Bojilov D., Mihailova G., Doncheva S. (2017). Antioxidant defense during desiccation of the resurrection plant *Haberlea rhodopensis*. Plant Physiol. Biochem..

[33] Farrant J.M., Jenks M.A., Wood A.J. (2007). Mechanisms of desiccation tolerance in angiosperm resurrection plants. Plant Desiccation Tolerance.

[34] Georgieva K., Mihailova G., Gigova L., Dagnon S., Simova-Stoilova L., Velitchkova M. (2021). The role of antioxidant defense in freezing tolerance of resurrection plant *Haberlea rhodopensis*. Physiol. Mol. Biol. Plants.

[35] Noctor G. (2006). Metabolic signalling in defence and stress: The central roles of soluble redox couples. Plant Cell Environ..

[36] Yamamoto H.Y., Bassi R., Ort D.R., Yocum C.F., Heichel I.F. (1996). Carotenoids: Localization and function. Oxygen Photosynthesis: The Light Reactions.

[37] Müller-Moulé P., Conklin P.L., Niyogi K.K. (2002). Ascorbate deficiency can limit violaxanthin de-epoxidase activity in vivo. Plant Physiol..

[38] Sgherri C., Stevanović B., Navari-Izzo F. (2004). Role of phenolics in the antioxidative status of the resurrection plant *Ramonda serbica* during dehydration and rehydration. Physiol. Plant..

[39] Augusti A., Scartazza A., Navari-Izzo F., Sgherri C.L.M., Stevanović B., Brugnoli E. (2001). Photosystem II photochemical efficiency, zeaxanthin and antioxidant contents in the poikilohydric *Ramonda serbica* during dehydration and rehydration. Photosynth. Res..

[40] Djilianov D., Ivanov S., Moyankova D., Miteva L., Kirova E., Alexieva V., Joudi M., Peshev D., Van den Ende W. (2011). Sugar ratios, glutathione redox status and phenols in the resurrection species *Haberlea rhodopensis* and the closely related non-resurrection species *Chirita eberhardtii*. Plant Biol..

[41] Toldi O., Tuba T., Scott P. (2009). Vegetative desiccation tolerance: Is it a goldmine for bioengineering crops?. Plant Sci..

[42] Jiang G., Wang Z., Shang H., Yang W., Hu Z., Phillips J., Deng X. (2007). Proteome analysis of leaves from the resurrection plant *Boea hygrometrica* in response to dehydration and rehydration. Planta.

[43] Cobbett C.S., Goldsborough P. (2002). Phytochelatins and metallothioneins: Roles in heavy metal detoxification and homeostasis. Annu. Rev. Plant Biol..

[44] Gilbert H.F. (1990). Molecular and cellular aspects of thiol-disulfide exchange. Adv. Enzymol. Relat. Areas. Mol. Biol..

[45] Furlan A., Bianucci E., Tordable M., Kleinert A., Valentine A., Castro S. (2016). Dynamic responses of photosynthesis and the antioxidant system during a drought and rehydration cycle in peanut plants. Funct. Plant Biol..

[46] Gołębiowska-Pikania G., Kopeć P., Surówka E., Krzewska M., Dubas E., Nowicka A., Rapacz M., Wójcik-Jagła M., Malaga S., Żur I. (2017). Changes in protein abundance and activity involved in freezing tolerance acquisition in winter barley (*Hordeum*
*vulgare* L.). J. Proteom..

[47] Sgherri C.L.M., Loggini B., Puliga S., Navari-Izzo F. (1994). Antioxidant system in *Sporobolus stapfianus*: Changes in response to desiccation and rehydration. Phytochemistry.

[48] Jovanović Ž., Rakić T., Stevanović B., Radović S. (2011). Characterization of oxidative and antioxidative events during dehydration and rehydration of resurrection plant *Ramonda nathaliae*. Plant Growth Regul..

[49] Veljović-Jovanović S., Kukavica B., Stevanović B., Navari-Izzo F. (2006). Senescence- and drought-related changes in peroxidase and superoxide dismutase isoforms in leaves of *Ramonda serbica*. J. Exp. Bot..

[50] Chandlee J.M., Scandalios J.G. (1983). Gene expression during early kernel developmental in *Zea mays*. Dev. Genet..

[51] Sherwin H.W., Farrant J.M. (1998). Protection mechanisms against excess light in the resurrection plants *Craterostigma wilmsii* and *Xerophyta viscosa*. Plant Growth Regul..

[52] Shivaraj Y., Plancot B., Gügi B., Kambalagere Y., Jogaiah S., Driouich A., Govind S.R. (2020). Vegetative desiccation tolerance in *Eragrostiella brachyphylla*: Biochemical and physiological responses. Heliyon.

[53] Kampfenkel K., Van Montagu M., Inze D. (1995). Extraction and determination of ascorbate and dehydroascorbate in plant tissues. Anal. Biochem..

[54] Griffith O.W. (1980). Determination of glutathione and glutathione disulfide using glutathione reductase and 2-vinylpyridine. Anal. Biochem..

[55] Mladenov P., Zasheva D., Djilianov D., Tchorbadjieva M. (2015). Towards proteomics of desiccation tolerance in the resurrection plant Haberlea rhodopensis. Compt. Rend. Acad. Bulg. Sci..

[56] Bradford M.M. (1976). A rapid and sensitive method for the quantitation of microgram quantities of protein utilizing the principle of protein-dye binding. Anal. Biochem..

[57] Laemmli U.K. (1970). Cleavage of structural proteins during the assembly of the head of bacteriophage T4. Nature.

[58] Azevedo R.A.D., Alas R.M., Smith R.J., Lea P.J. (1998). Response of antioxidant enzymes to transfer from elevated carbon dioxide to air and ozone fumigation, in the leaves and roots of wild-type and a catalase-deficient mutant of barley. Physiol. Plant..

[59] Anderson M.D., Prasad T.K., Stewart C.R. (1995). Changes in isozyme profiles of catalase, peroxidase, and glutathione reductase during acclimation to chilling in mesocotyls of maize seedlings. Plant Physiol..

[60] Ricci G., Bello M.L., Caccuri A.M., Galiazzo F., Federici G. (1984). Detection of glutathione transferase activity on polyacrylamide gels. Anal. Biochem..

